# AI Radar Sensor: Creating Radar Depth Sounder Images Based on Generative Adversarial Network

**DOI:** 10.3390/s19245479

**Published:** 2019-12-12

**Authors:** Maryam Rahnemoonfar, Jimmy Johnson, John Paden

**Affiliations:** 1 Computer Vision and Remote Sensing Laboratory, University of Maryland, Baltimore, MA 20742, USA; 2 Department of Computing Sciences, Texas A&M University-Corpus Christi, Corpus Christi, TX 78412-5774, USA; jay4842@gmail.com; 3 Department of Center for Remote Sensing of Ice Sheets, University of Kansas, Lawrence, KS 66045, USA; paden@ku.edu

**Keywords:** convolutional neural network, generative adversarial network, ice tracking, radar imagery

## Abstract

Significant resources have been spent in collecting and storing large and heterogeneous radar datasets during expensive Arctic and Antarctic fieldwork. The vast majority of data available is unlabeled, and the labeling process is both time-consuming and expensive. One possible alternative to the labeling process is the use of synthetically generated data with artificial intelligence. Instead of labeling real images, we can generate synthetic data based on arbitrary labels. In this way, training data can be quickly augmented with additional images. In this research, we evaluated the performance of synthetically generated radar images based on modified cycle-consistent adversarial networks. We conducted several experiments to test the quality of the generated radar imagery. We also tested the quality of a state-of-the-art contour detection algorithm on synthetic data and different combinations of real and synthetic data. Our experiments show that synthetic radar images generated by generative adversarial network (GAN) can be used in combination with real images for data augmentation and training of deep neural networks. However, the synthetic images generated by GANs cannot be used solely for training a neural network (training on synthetic and testing on real) as they cannot simulate all of the radar characteristics such as noise or Doppler effects. To the best of our knowledge, this is the first work in creating radar sounder imagery based on generative adversarial network.

## 1. Introduction

Ice loss in Greenland and Antarctica has accelerated in recent decades. Melting polar ice sheets and mountain glaciers have considerable influence on sea-level rise (SLR) and ocean currents; potential floods in coastal regions could put millions of people around the world at risk. Precise calculation of ice thickness is very important for sea level and flood monitoring. The shape of the landscape beneath the thick ice sheets is an important factor in predicting ice flow. Radars are one of the most important sensors that can penetrate through ice and give us information about the ice thickness.

Several semi-automated and automated methods exist for layer finding and estimating ice thickness in radar images [1,2,3,4,5,6]. Crandall et al. [1] used probabilistic graphical models for detecting the ice layer boundary in echogram images from Greenland and Antarctica. The extension of this work was presented in [2] where they used Markov-chain Monte Carlo to sample from the joint distribution over all possible layers conditioned on an image. Mitchell et al. [3] used a level set technique for estimating bedrock and surface layers. However, for every single image, the user needs to re-initialize the curve manually and as a result, the method is quite slow and was applied only to a small dataset. This problem was fixed in [4,5], where authors introduced a distance regularization term in the level set approach to maintain the regularity of level set intrinsically. Therefore, it does not need any manual re-initialization and was automatically applied to a large dataset. However, their technique has difficulty in detecting the ice bottom when it is faint. The issue of detecting faint layers was improved in [6] based on an electric charged particle concept. The main issue with the current techniques is that they are based on feature engineering techniques, which means a set of parameters need to be defined and tuned for each case and cannot be scaled to big data.

In recent years, the research community has witnessed advances in artificial intelligence (AI). Recent advances in deep neural networks (DNNs) and massive datasets have facilitated progress in AI tasks such as classification [7,8,9,10], object recognition [11,12], counting [13,14,15], contour and edge detection [16] and semantic segmentation [17,18,19]. Despite this progress, these algorithms are limited to cases where large labeled datasets are available.

The vast majority of data available in the remote sensing community is unlabeled, and the labeling process is both time-consuming and expensive. One possible alternative to the labeling process is the use of synthetically generated data. The standard way for generating synthetic radar data (such as from a radar depth sounder used to measure ice thickness) is to simulate the radar scattering response using digital elevation models (DEM) of the ice surface and bottom. Usually, the DEM is represented by a sheet of points or facets and the total scattering response is the superposition of the scattering from all of these targets. This data can then be processed through the regular radar data processing chain to produce a simulated radar image. However, data generation based on a physics simulation is compute-intensive and cannot be used for generating large data sets.

**Figure 1:**
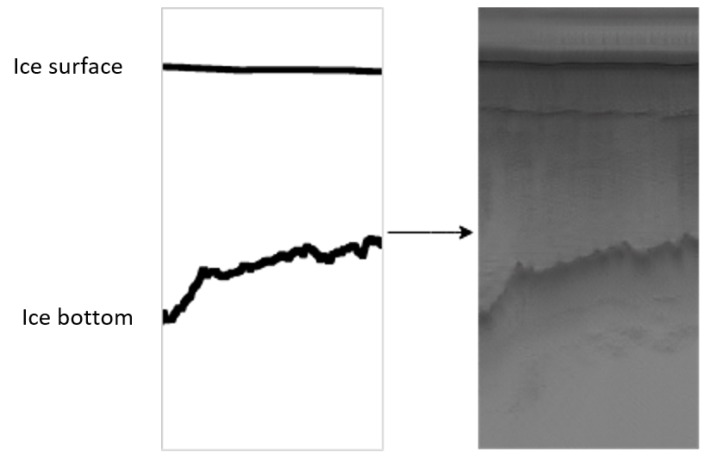
Generated radar depth sounder image (**right**) from arbitrary polynomials representing the surface and bottom of the ice (**left**). The horizontal axis is the flight path and the vertical axis is the ice depth.

The goal of this research is to generate synthetic radar images that can be used to train data-driven algorithms such as deep convolutional neural nets. By synthetic radar image, we mean a radar image that is generated by data-driven methods without any knowledge of the physics of the sensor or the environment. In other words, our synthetic radar image simulates the appearance of real radar imagery. Using some random polynomials that represent the bottom and surface of the ice, we can generate synthetic radar images that correspond to each label. In this way, we can generate unlimited pairs of labels and images. In this research, we developed a data-driven machine learning approach, Generative Adversarial Networks (GANs), for generating synthetic radar data. A GAN [20] is composed of two simultaneously trained parts called a generator and discriminator. The discriminator is trained to tell the difference between real and fake images. The generator is trained to generate realistic-looking images and fool the discriminator. Both components improve until the synthetic images are indistinguishable from the real images. The discriminators accuracy reduces to 0.5, indicating that is simply guessing when it makes its decision. For generating synthetic radar images from labeled data, contours showing the ice surface and ice bottom, (Figure 1-left), we adopted the CycleGAN framework [21] over other image to image translation based GAN’s primarily due to the standard static architecture, it’s ability to train with unpaired images, performance, and its ability to handle texture changes well. Figure 1(right) shows the synthetic radar image which is generated from an arbitrary polynomial representing ice surface and bottom.

The CycleGAN network works with two different sets of images. Each set of images has its own discriminator. Two different mapping functions are used, called G and F. Each mapping function translates an image from one set to the other. The network works with the intuition that if an image from one set is translated to the other, and that the resulting image is translated back to the original set, the final result should be approximately the same as the original. The difference in these images is termed cycle-consistency loss, and is what the network tries to minimize.

Our contributions are summarized as followings:

- Generating synthetic radar images and their corresponding labels based on modified cycle-consistent adversarial networks.
- Testing and evaluation of generated synthetic imagery based on both qualitative and quantitative similarity indexes.
- Testing of the generated images for data augmentation and training of a contour detection algorithm.
- Collecting a novel data set of radar imagery from the Arctic and Antarctic.

To the best of our knowledge, this is the first work in creating radar sounder imagery based on generative adversarial network.

## 2. Related Work

Generative adversarial networks (GANs) originally proposed by Goodfellow [20] are comprised of two separate networks called the generator and discriminator. These two components are trained in a competitive framework, with each learning the features of the input image data set. The task of the discriminator is to learn the difference between real and fake images, while the generator attempts to generate realistic images that can fool the discriminator. GANS can be distinguished by the type of input used as the seed for generating the output. Radford et al. [22] presented a network referred to as deep convolutional GAN (DCGAN) where the authors emphasize the use of stridden convolutions over pooling layers, the use of batch normalization, the removal of fully-connected layers, and the use of ReLU and leaky ReLU activation functions. In paired input GANs, the random noise input is replaced with a conditional image. Extending the framework of the DCGAN, Conditional GANs (cGANs) [23] use input in the form of an edge map, semantic label, or gradient field. The cGAN allows for “translation” of one representation of an image to another. Experiments shown in the work include translating between maps and aerial photos, black and white image to color image, and architectural labels to photo. The limitation of needing paired input can be addressed by using other GAN implementations that work with unpaired input. In another extension to the DCGAN framework, CycleGAN [21] learns features from two input datasets at the domain level. Two mapping functions are used that allow translation to happen in a bidirectional manner. After translating an image to a different domain, the additional mapping function is also used to “reconstruct” the original image. The difference between these images is the basis for the “cycle-consistency loss” which is used to train the network. Recent works have built on the ideas introduced by CycleGAN to suite their problem and dataset [24,25,26,27,28,29]. Similar to the idea of building of the CycleGAN we modify the cycle consistency portion of the loss function to suit our problem.

GANs are notorious for being difficult to train; when training a GAN we have to focus on both the generator and discriminator. While training they both exploit the modulations in the data. This ultimately results in a mode collapse of either the generator or the discriminator [30,31,32,33]. Mode regularization [30] takes a look into the issue of GANs sensitivities to diminishing gradients by introducing ways to regularize GANs through regularization, manifold-diffusion training, and a mode missing metric [30]. CycleGAN focuses on assessing mode collapse by using a history of generated data to be used in the discriminator [21,34].

GANs have recently been used in many applications including generating images of buildings from a facade mask, road maps from aerial images, visible imagery from SAR imagery, and multi-sensor data fusion [21,23,35,36,37]. In [37], the cGANs framework was used to generate artificial templates to help with the problem of multi-sensor data fusion, dealing specifically with optical and SAR images. Using cGANs, the feature extraction step usually needed for template generation can be skipped. Artificial SAR-like templates were generated from the optical images and used to increase the matching accuracy of both similarity-based and feature-based approaches. In [36] a single-channel noisy SAR image was mapped into a visible-like RGB image using GANs. Here we generate a novel dataset of synthetic Radar depth Sounder Imagery of Ice sheets based on the arbitrary polynomials of ice surface and bottom boundaries.

GANs have also been used for data augmentation in several cases [38,39,40,41]. In [38], the DCGAN framework was used to supplement a dataset of computed tomography (CT) images of liver lesions. The enriched dataset was used to train a classification CNN to determine the specific type of lesion from three categories. The experiment showed that the synthetic images allowed the classifier to achieve better performance beyond the previous maximum. A similar use can be seen in [39] where positron emission tomography (PET) images were synthesized from CT images. Conditional GANs have been used to generate synthetic images of plants given an input mask. The generated images were used to combat a scarcity of data for phenotyping problems [40]. Similar to this work there is also a scarcity of mammogram classification datasets mainly due to privacy concerns. A type of conditional GAN was used to generate malignant and non-malignant images using Infilling [41]. In this work, we compare multiple dataset samples to assess how a contour detection network is improved by introducing augmented data generated by GAN.

## 3. Materials And Methods

### 3.1. Formulation

**Figure 2:**
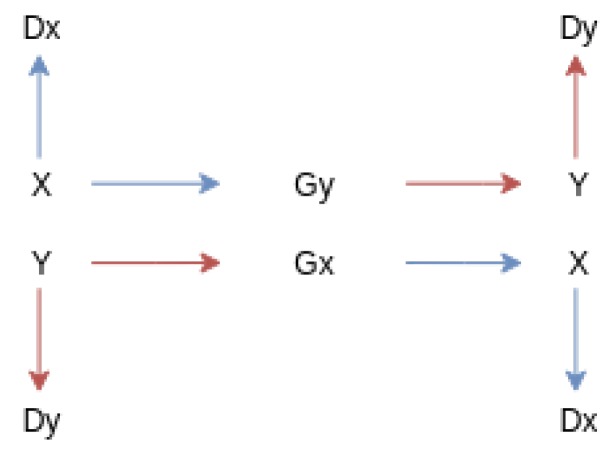
Starting with an input image that is fed into a generator either $G_{x}$ or $G_{y}$, we can map that input to the desired output. This output can then be fed into the opposite generator to return back to the original domain.

The initial goal inherited from the CycleGAN is to learn the mapping functions between the two domains X and Y [21] (Figure 2). In the first stage, adversarial loss [20] is applied to asses each generator that will be responsible for learning one domain: G:X→Y and F:Y←X. Additionally each mapping function will also be paired with a discriminator $D_{X}$ and $D_{Y}$. The adversarial loss [20] for the mapping function can expressed as [21]:

(1) $$L_{GAN}\left(G,D_{Y},X,Y\right)=E_{y∼pdata}\left(y\right)\left[logD_{Y}\left(y\right)\right]+E_{x∼pdata}\left(x\right)\left[log\left(1-D_{Y}\left(G\left(x\right)\right)\right)\right],$$

where *G* attempts to generate images G(x) that look similar to the images of domain *Y*, while $D_{Y}$ will try to discriminate between the translated samples G(x) and the real samples *y*. *G*’s goal is to minimize this objective against its adversary *D* while *D* will aim to maximize it. Using this we can also apply it to another mapping function F:Y→X and its discriminator $D_{X}$ [21].

Using the adversarial loss these networks can, in theory, learn mappings *G* and *F* that produce outputs identical to the target domains *X* and *Y* if *G* and *F* are stochastic functions [42]. However, with large enough capacity, a network can map the same set of input images to any random permutation of images in the target domain, where any of the learned mappings can induce an output distribution that matches the target distribution. The cycle constancy loss is defined according to the following formula:

(2) $$L_{cyc}\left(G,F\right)=E_{x∼pdata}\left(x\right)[∥F\left(G\left(x\right)\right)-x∥]+E_{y∼pdata}\left(y\right)[∥G\left(F\left(y\right)\right)-y∥].$$

Here we have used a more numerically stable function (L2 loss) comparing to L1 loss. A comparison between L1 and L2 loss is discussed further in the result section.

The full objective that is inherited from CycleGAN [21] is the combination of the two losses with an addition of the λ parameter that control importance of the losses.

(3) $$L\left(G,F,D_{X},D_{Y}\right)=L_{GAN}\left(G,D_{Y},X,Y\right)+L_{GAN}\left(F,D_{X},Y,X\right)+\lambda L_{cyc}\left(G,F\right).$$

Each of the GAN losses apply mean squared error as the criterion. Training the *G* to minimize $E_{x∼pdata}\left(x\right){\left[D\left(G\left(x\right)\right)-1\right]}^{2}$ and train the *D* to minimize $E_{y∼pdata}\left(y\right){\left[D\left(y\right)-1\right]}^{2}+E_{y∼pdata}\left(x\right){\left[D\left(G\left(x\right)\right)\right]}^{2}$ [21]. This proves to be suitable for images that have larger values or have less discrete values. Additionally, it is also a suitable criterion for generalization.

### 3.2. Network

**Figure 3:**
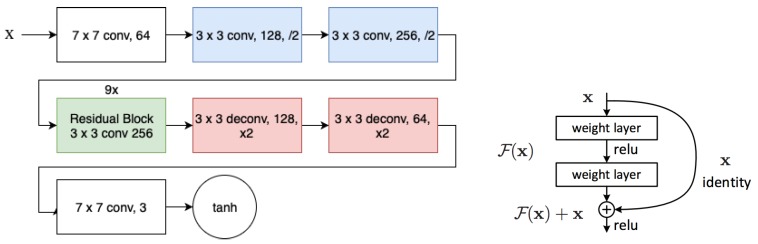
Architecture of the generator network (**left**). Residual block [43] is the main component of generator network (**right**).

The architecture of the generator network is depicted in Figure 3. This network includes two stride of two convolutions and nine residual blocks [43] followed by two deconvolution layers. Additionally each convolution is also activated by a rectified linear unit (ReLu).

**Figure 4:**
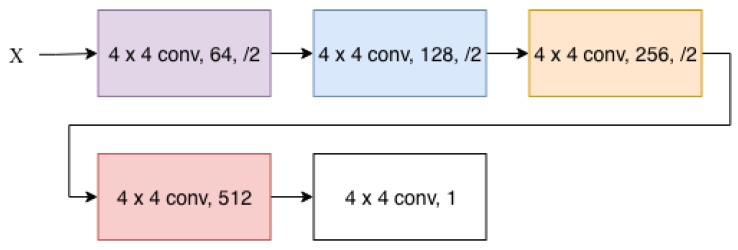
Architecture of the discriminator network.

For the discriminator we maintain the network used by CycleGAN [21] which is a convolutional neural network with five convolutions. The discriminator network is shown in Figure 4. Additionally, each operation is also followed by an instance normalization [44]. Using these residual connections shows higher stability in unpaired image to image translation, versus a U-net type network with the skip connections based on an encoding and decoding pathway. This can lead to instability with unpaired training due to less information being available.

**Figure 5:**
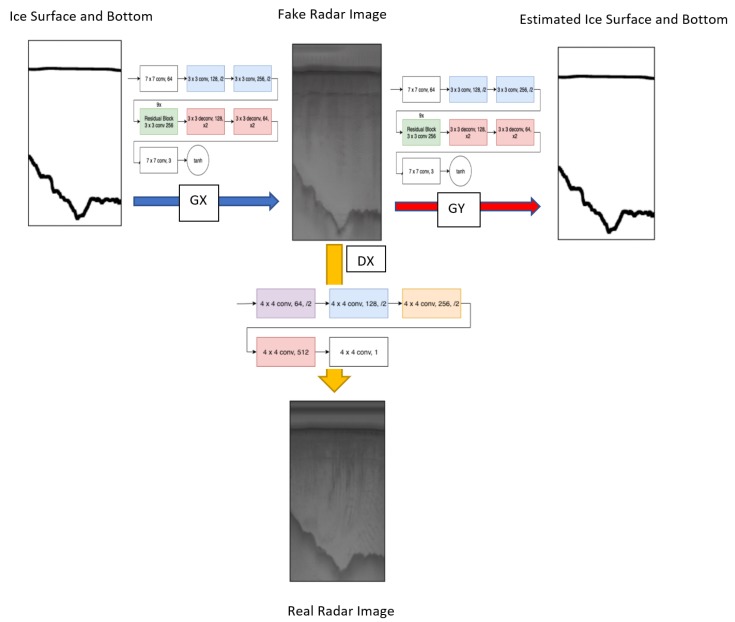
Flowchart of generating synthetic radar images from ice surface and bottom layer data. F and GY are the generative networks while DX is the discriminator network.

We also use Shrivastava’s strategy [34] to reduce model oscillation [42] by updating the discriminators using a history of generated output. This allows the discriminator to view past generated images that may look worse than a newly generated image; giving the discriminator a sort of memory [34]. This generated output is stored in an image pool that will fill until it has reached its maximum size. After that size is reached then the images that have been stored will be swapped with newly generated images as more images are generated. For training we test different values for hyperparameters and based on our experimental results we set λ=10.0 for both domains, use the Adam Optimizer [42] with a learning rate of 0.0002. The overall flowchart of our method is shown in Figure 5.

## 4. Experimental Results

### 4.1. Dataset

The images used in this research are CReSIS standard output products collected with a radar depth sounder (RDS) from the years 2009 to 2017. The horizontal axis is along the flight path and the vertical axis represents depth. The dark line on the top of the image is the boundary between air and ice while the more irregular lower boundary represents the boundary between the ice and the terrain.

To train the CycleGAN network we used the RDS dataset and the ground-truth images which are produced by human annotators. The RDS dataset includes images of various sizes. These images were then split up into slices which are 512 by 1024 pixels. For the sake of this network, the input images are downsized to 128 by 256 pixels. Our dataset is comprised of 20,463 training images and 8769 testing images and the ground-truth images (boundary layers) associated with them.

To test and evaluate the performance of CycleGAN approach we conducted several experiments including (1) extracting labels from the real radar images using mapping function Y, (2) generating synthetic images from the ground-truth labels using mapping function G (3) generating synthetic images from arbitrary polynomial labels and (4) testing and evaluation of state-of-the-art contour detection algorithm for detecting labels on the synthetic radar images generated by CycleGAN and its combination with real data.

### 4.2. Qualitative Results

**Figure 6:**
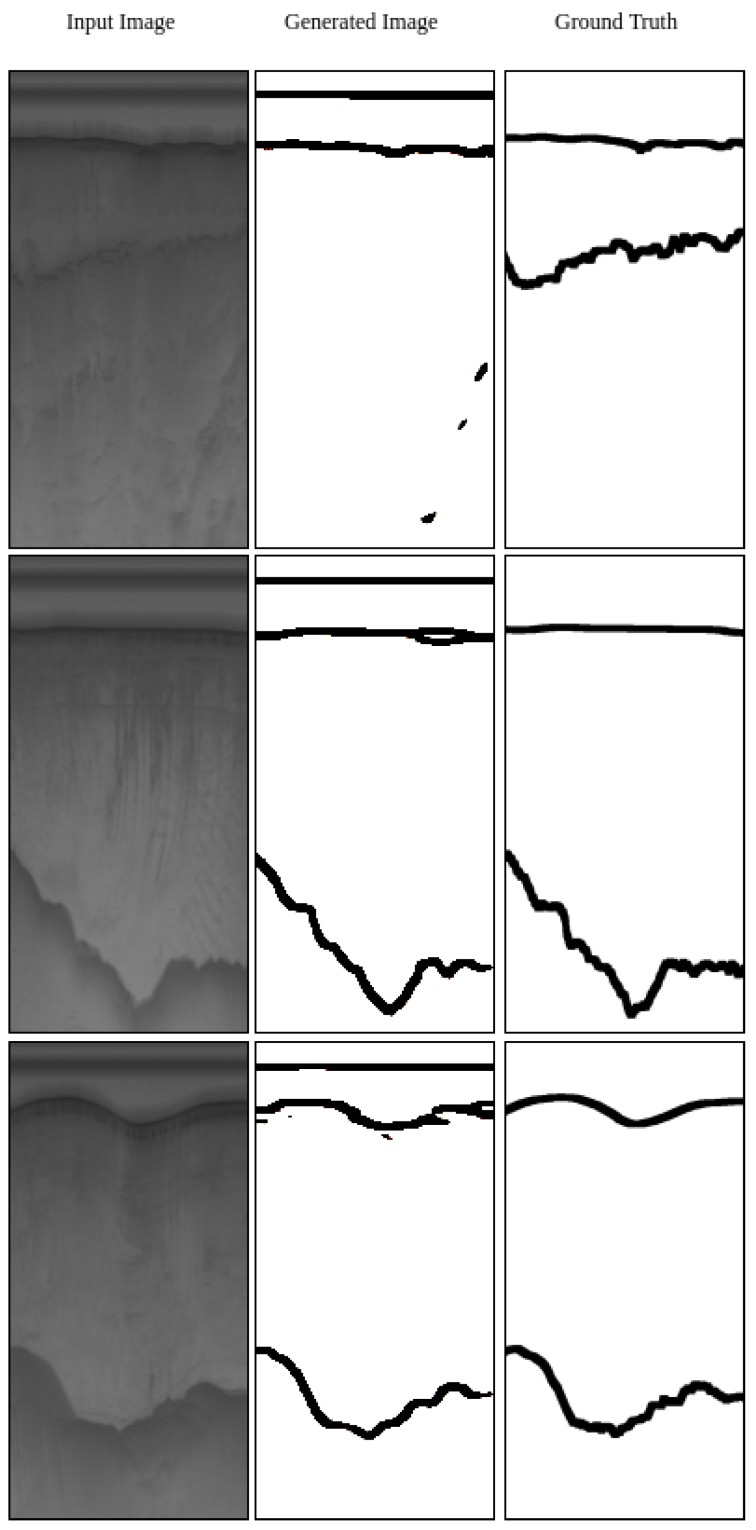
Image to label (Y) results paired with the actual labels (**right**) for comparison. The input to the network is the image (**left**) while it generates the label (**middle**).

**Figure 7:**
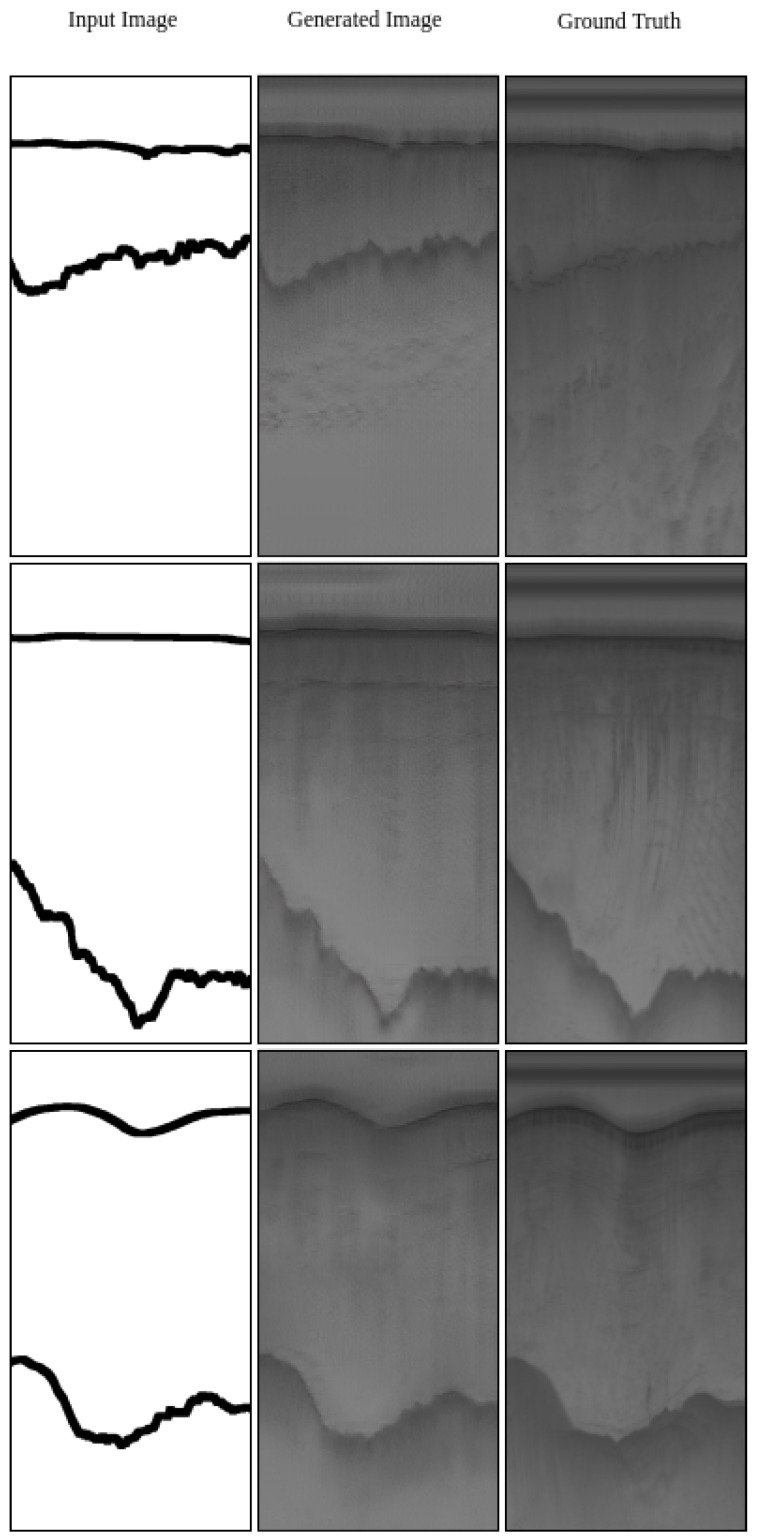
Label to Image (G) results paired with the actual radar image (**right**) for comparison. The input to the network is the label (**left**) while it generates the image (**middle**).

The result of generating labels from the real radar data and also generating synthetic images from ground-truth labels are displayed in Figure 6 and Figure 7, correspondingly.

Figure 6 shows that overall the algorithm can generate correct labels. The left column in Figure 6 shows the real radar images, the second column shows the labels generated by Generator *Y* and the right column shows the corresponding ground-truth labels. As we can see in the Figure, when there is a direct path signal at the top of the image, Generator *Y* detects both the direct path and the actual ice surface. Moreover, when there is a faint bottom layer, the generator is not able to detect it well (Top row in Figure 6).

Figure 7 shows that mapping function *G* can generate high-quality synthetic radar images. In this figure, the inputs to the algorithm are ground-truth labels (left column). The Generator *G* generated the synthetic images using the input labels (middle column). The generated synthetic images look very much like real radar images (right column). It only struggles with minor details; for example, it cannot generate the direct path signal at the top of the image.

**Figure 8:**
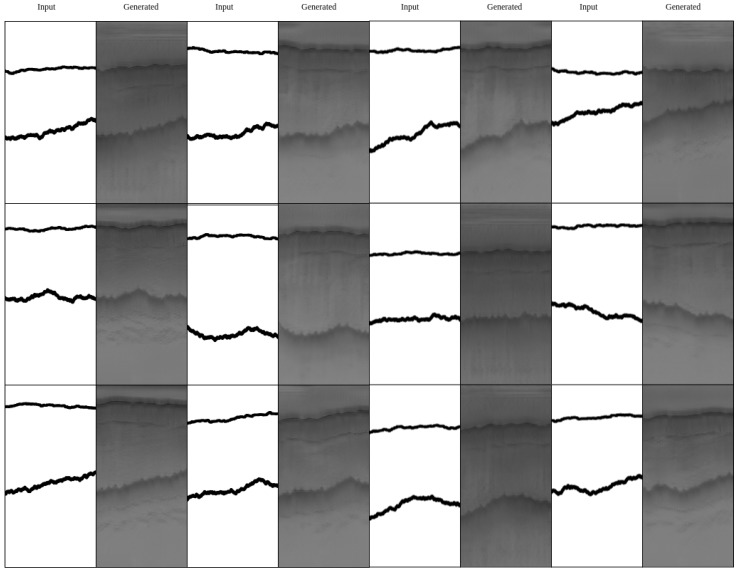
Fully synthetic images generated by G(x) by feeding in randomly generated ground truth labels. Observing these results, we can see that the generative adversarial network (GAN) produces synthetic images that resemble the structure of original images. Additionally, these results show that we can use this model for further data augmentation.

In addition to generating radar images from actual labeled data, we also generated radar images from arbitrary labels. Using the idea of tracing an image, we created lines by selecting a starting point at random position and then for the top line moving along the x-axis and changing the y coordinate value by one. This slight change keeps the deviation slight for the top line. Secondly, the bottom line contains a more visible deviation. Similar to the top line, we select a random point to start drawing our line at a random position. The only difference is that the y coordinate will change by two versus one. Figure 8 displays several images that were generated by G(x) with synthetic labels as the input. This shows that we are able to generate fully-synthetic images using the mapping function G(x). This process can help in data augmentation of deep neural networks when labeled data are not available.

### 4.3. Quantitative Results: Survey

To evaluate the quality of the images that are generated by our algorithm, we conducted a survey in addition to calculating similarity matrices. In the survey, we first displayed real images to 20 individuals showing the respondent what a real ice image looks like. After that, they were asked to view 20 images and determine if the image in front of them was real or fake. The results showed that out of 20 images (10 real and 10 fake) they were collectively unable to determine if the images were real or fake. In average they scored 50.18% of images as real and 49.81% as fake.

### 4.4. Quantitative Results: Similarity Metrics

Along with the survey, we also include two other metrics namely structural similarity index (SSIM) [45,46,47] and peak signal to noise ratio (PSNR).

The structural similarity index is expressed as [45,46]:

(4) $$SSIM\left(x,y\right)=I{\left(x,y\right)}^{\propto }C{\left(x,y\right)}^{\beta }S{\left(x,y\right)}^{\gamma },$$

where *I* is luminance, *C* is contrast, and *S* is structure [45,46]. The SSIM attempts to model the structural change of an image by comparing small windows or sub-samples in the image to compare the luminance, contrast, and structure of the two images [46]. This metric gives us a robust measure of the perceived changes in the image. The closer the SSIM is to 1.0 the higher the quality image we have [46,48]. Another evaluation metric is peak signal to noise ratio (PSNR) or signal to noise ratio (SNR) [49,50] which is commonly used in the signal processing area as an image quality metric. PSNR is expressed as [51]:

(5) $$PSNR=10log_{10}\frac{255^{2}}{\langle n{\left(x,y\right)}^{2}\rangle },$$

where $\langle n{\left(x,y\right)}^{2}\rangle$ gives mean square error [51]. The higher the PSNR (in dB), the better the quality of the generated image [48]. Using these two metrics we can show how well the generated synthetic images look like the real images. In our experiment, the average SSIM is equal to 0.824 and the average PSNR is equal to 25.726dB. The SSIM value is close to 1 which means two sets of images are very similar. PSNR is usually around 20 dB for the visible images generated by GAN algorithm. PSNR for Radar imagery is usually between 25–30 dB. This shows that our generated images have similar noise content and comparable image quality to real radar imagery.

### 4.5. Cycle Loss Evaluation

To display how using L2 loss versus L1 loss affected our experiment we ran a number of experiments to show how changing the cycle consistency loss and also hyperparameters can affect the network’s performance. We performed extensive testing on a small dataset for selecting our loss function and hyperparameters. Overall L2 loss (MSE loss) performed better for all hyperparameters as it can be seen in Table 1 and Table 2.

On a small dataset, we tested different combinations of hyperparameters which both λ varied between 5–15, β between 0.3–0.7 and learning rate between 0.00002–0.002. On a small dataset and with L2 loss function the following combinations of hyperparameters show the best performance which we finally tested on our entire large dataset.

In Table 3 the first two columns (SSIM and PSNR) evaluate the quality of generated images while the rest of the columns evaluate the quality of generated labels.

### 4.6. Quantitative Results: Improving Edge Detection

One of the goals of creating additional synthetic images through a GAN is to attempt to improve the performance of contour detection algorithms [47,52,53,54]. Deep learning approaches are data hungry; providing a large dataset of labeled data is both time-consuming and expensive. Through training with synthetic data along with real data, we hypothesize that this training method will improve the results comparing to training with only real data. The reason behind it is not only increasing the number of training images but also augmenting quality images. Our synthetic images are less noisy comparing to the real images.

To approach this experiment we used holistically-nested edge detection (HED) [52]. The HED uses the VGG16 architecture with the addition of five side outputs to provide multi-scale images and multi-scale feature learning. The HED is composed of five convolutional blocks each containing a number of convolutions. At the end of each convolutional block, there is a side output that consists of a 1 × 1 convolution followed by deconvolution to upsample the output to match the input size. Then once the five side outputs are produced they are concatenated and then fused together by a 1 × 1 convolution [52].

There are two image sets, real and synthetic and they both contain the same number of images. There were six experiments conducted to evaluate the effectiveness of adding the additional data. The first two experiments are the real and synthetic datasets meaning that the HED algorithm was trained and tested on real data, and synthetic data, correspondingly. The next four experiments use various dataset combinations. Mixture 1 for training uses the equal number of real and synthetic images for training (20,463 real and 20,463 synthetic images) and an equal number of real and synthetic data for testing (8769 real images and 8769 synthetic images). Mixture 2 for training uses the real training images and half of the synthetic training images. For testing uses the real testing images and the synthetic testing images. Mixture 3 for training uses the synthetic training images and for testing uses the real testing images. Mixture 4 for training uses half of the real training images and the synthetic training images and for testing uses the real testing images. Table 4 shows the number of the images used for training and testing at each experiment and also the results of the six experiments.

As displayed in Table 4, Mixture 1 has the highest F scores; It shows that adding synthetic images will increase the performance of the edge detection algorithm. Adding more synthetic images during training will increase the performance. That is the reason that mixture 1 has higher accuracy comparing to mixture 2. However, mixture 3 has the lowest F scores as it is trained on synthetic data but tested in real images. Therefore it is important that the algorithm sees some sample of real data during the training. When we add half of real training data to synthetic training data but still test it on real data (mixture 4), F scores are improved comparing to mixture 3. F-scores of mixture 4 are higher than when we train and test on synthetic images (compare synthetic row with mixture 4 row) and F2 score is even higher than the training and testing on real images.

In general, there is an improvement to the network once we introduce synthetic examples. F-scores are increased by introducing the synthetic training examples along with the real data (mixture 1). Based on our observations, although GANs generate synthetic images that look very much like real data, they cannot simulate all radar characteristics and that is why training on synthetic and testing on real data (mixture 3) cannot produce high accuracy results. The reason that mixture 1 has the highest F-score is that there is an equal distribution of real and synthetic images during both training and testing while mixture 4 is mainly dominated with synthetic images during training.

**Figure 9:**
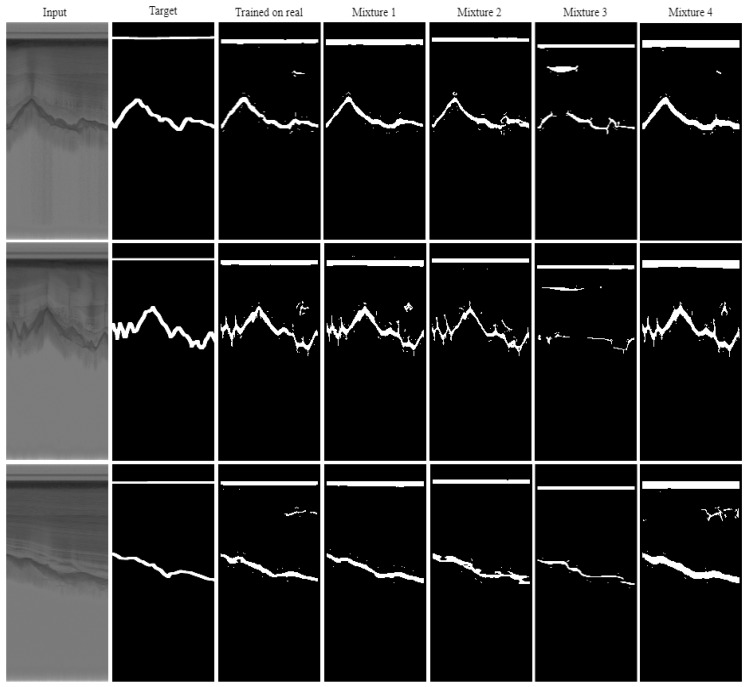
HED results after training with the six dataset splits. These results include real image examples.

**Figure 10:**
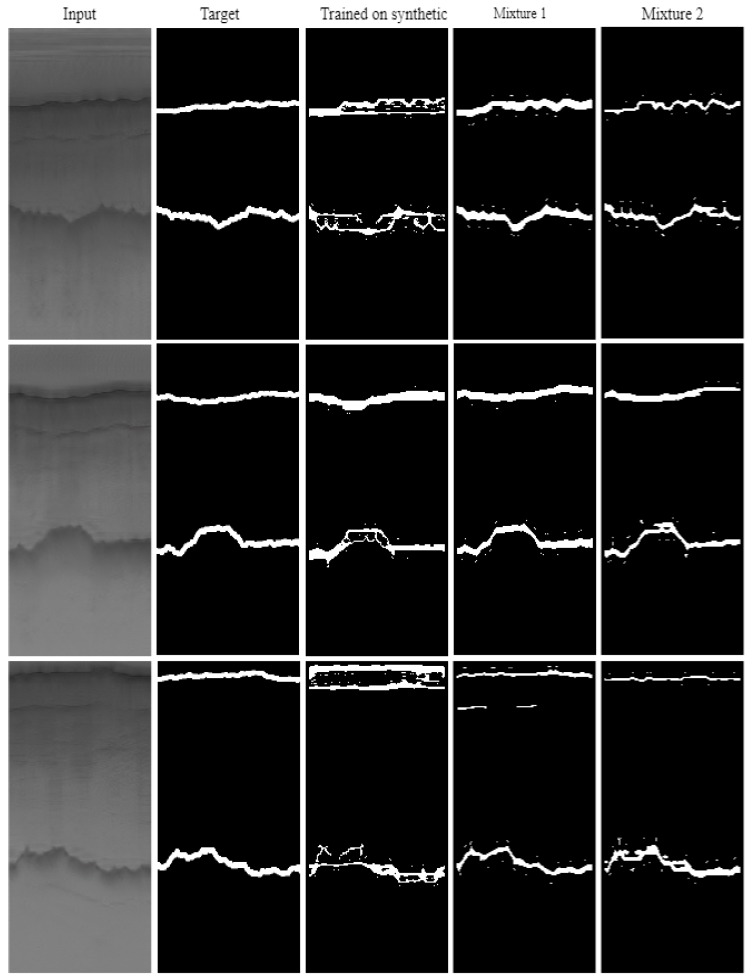
HED results after training with the six dataset splits. These results include synthetic data during both training and testing.

Figure 9 and Figure 10 shows some qualitative results. As can be seen in this figure, mixture 1 that has the highest number of synthetic images during the training has the highest quality while mixture 3 which is trained on synthetic images and tested on real images, has the lowest quality.

## 5. Conclusions

Here we developed an architecture based on the CycleGAN network to generate synthetic radar depth sounder images. This method can also be used to generate other types of radar images. We conducted several experiments for testing the generated synthetic images based on qualitative survey and qualitative similarity metrics. Based on qualitative survey users were not able to distinguish between real and simulated data. Similarity metrics also demonstrate high statistical proximity of AI-generated results to the real radar data. We also tested the performance of a contour detection algorithm based on different combinations of real and synthetic data. Our experiments show that synthetic radar images generated by GANs can be used in combination with real images for data augmentation and training of deep neural networks. However, the synthetic images generated by GANs cannot be used solely for training a neural network because they cannot simulate all of the radar characteristics such as noise or Doppler effects. This shows that our simulated data are very similar to real data from the appearance and statistical point of view but the model cannot simulate the physics. Moving forward we plan to apply the simulated data generation to snow radar data that contain more than two layers. This will also lead us to develop a stronger label creator that can take more factors into consideration such as the geometric shapes of layers. We will also explore the combination of AI and physics simulators for a more realistic radar data simulator. 

## Figures and Tables

**Table 1:** Comparison between L1 and L2 cycle loss function on different image quality metrics for a small dataset.

Loss Function	SSIM (Avg)	SSIM (Min)	SSIM (Max)	PSNR (Avg)	PSNR (Min)	PSNR (Max)
L1 Cycle Loss [21]	0.68	0.016	0.79	19.15	4.67	24.44
L2 Cycle Loss	0.72	0.57	0.8	20.93	17.78	23.32

**Table 2:** Comparison between L1 and L2 cycle loss function on different edge detection metrics for a small dataset.

Loss Function	Precision	Recall	F1	F2
L1 Cycle Loss [21]	0.007	0.008	0.007	0.008
L2 Cycle Loss	0.04	0.1	0.04	0.048

**Table 3:** Comparison between different hyperparameters on our entire dataset. These hyperparameters are chosen after extensive test with different combinations of hyperparameters on small dataset.

Hyper Parameters	SSIM	PSNR	Precision	Recall	F1	F2
$\lambda_{A}=5$, $\lambda_{B}=5$, β=0.7, R = 0.0002	0.75	23.69	0.048	0.058	0.05	0.06
$\lambda_{A}=10$, $\lambda_{B}=10$, β=0.5, R = 0.0002	0.82	25.71	0.33	0.49	0.38	0.43

**Table 4:** Comparison between training an holistically-nested edge detection (HED) [52] with six types of datasets.

Dataset Used	Number of Train/Test Samples	Precision	Recall	F1 Score	F2 Score
Real	20,463/8769	0.518	0.586	0.507	0.534
Synthetic	20,463/8769	0.417	0.500	0.451	0.478
Mixture 1	40,926/17538	0.575	0.660	0.590	0.621
Mixture 2	30,694/17538	0.522	0.528	0.506	0.510
Mixture 3	20,463/8769	0.172	0.136	0.139	0.134
Mixture 4	30,694/8769	0.394	0.680	0.463	0.551
